# Accumulation and Elimination of Enrofloxacin and Ciprofloxacin in Tissues of Shrimp *Litopenaeus vannamei* under Laboratory and Farm Conditions

**DOI:** 10.5402/2012/374212

**Published:** 2012-06-16

**Authors:** Brisa Marisol Flores-Miranda, Angelica Espinosa-Plascencia, Silvia Gómez-Jiménez, Alonso Alexis López-Zavala, Haydé Hayamaí González-Carrillo, María del Carmen Bermúdez-Almada

**Affiliations:** Research Center for Food and Development AC, Coordination of Food Science, Carretera a la Victoria Km. 0.6, 83000 Hermosillo, SON, Mexico

## Abstract

This study aimed to quantify the accumulation and elimination of Enrofloxacin (ENRO) and Ciprofloxacin (CIPRO) in cultivated *Litopenaeus vannamei* under controlled laboratory and farm conditions. Laboratory- and farm-raised shrimp were given feed supplemented with 200 mg/kg ENRO for 14 days, followed by a 16-day diet without antibiotics. The levels of ENRO and CIPRO were analyzed by High Performance Liquid Chromatography (HPLC). In the laboratory, ENRO concentrations in the muscle and hepatopancreas reached a maximum (C_max_) of 0.54 ± 0.26 **μ**g/g and 3.52 ± 1.9 **μ**g/g, respectively; C_max_ values for CIPRO in the laboratory were 0.18 ± 0.13 **μ**g/g (muscle) and 1.05 ± 0.20 **μ**g/g (hepatopancreas). In farmed shrimp, C_max_ values for ENRO were 0.36 ± 0.17 **μ**g/g muscle and 1.60 ± 0.82 **μ**g/g in the hepatopancreas; CIPRO C_max_ values were 0.03 ± 0.02 **μ**g/g (muscle) and 0.36 ± 0.08 **μ**g/g (hepatopancreas). Two to fourteen days were necessary to eliminate both antibiotics from muscular tissue and four to more fourteen days for complete elimination of the antibiotics from the hepatopancreas. These results should be considered in terms of minimum concentrations necessary to inhibit *Vibrio* bacteria to determine whether the current use of this antibiotic is effective in controlling disease.

## 1. Introduction

One alternative to treating bacterial infections in aquaculture is to use antibiotics during cultivation, as is common in shrimp aquaculture. Enrofloxacin, oxytetracycline, and florfenicol are among the most commonly used antibiotics during shrimp cultivation and are commonly mixed into food pellets; however, the inappropriate use of these compounds can result in accumulation of residual antibiotic in tissue and contribute to the emergence of resistant bacteria via residual antibiotics persisting in the sediment.

ENRO is a fluoroquinolone (FQ), nalidixic acid derivative with broad-spectrum activity against Gram-negative bacteria. The core structure is a dihydroquinoline or 4-quinolone ring; this structure is lipophilic and has a low molecular weight, promoting tissue penetration [1]. ENRO inhibits bacterial DNA gyrase, preventing DNA synthesis [2]. Although the mechanism of action is known, the pharmacokinetics and resultant bioavailability of this antibiotic in shrimp aquaculture are poorly understood. More data are needed to determine the optimal drug dosage for use in shrimp farming and to establish the levels of ENRO accumulation in organs. This knowledge is crucial to establish the presence of residues in shrimp tissue, thus reducing the risks of sediment accumulation and emerging bacterial resistance.

Studies have been done on the pharmacokinetics of ENRO and CIPRO in other species, such as, crab (*Scylla serrata*), fish (*Oreochromis niloticus*), black shrimp (**Penaeusmonodon**), Chinese shrimp (*Penaeus chinensis*), and bass (*Dicentrarchus labrax*) [3–6]. These results, however, should not be extrapolated to **L*.  *vannamei**, as the metabolic differences between species and the environmental conditions in which an organism grows can influence on the kinetic behavior of ENRO.

The recent growth of shrimp farming in northwestern Mexico has created a need for research on the safe and effective use of ENRO in shrimp aquaculture. To that end, this study aims to determine in vivo, under controlled conditions (in a laboratory) and semicontrolled conditions (on a shrimp farm), the accumulation and distribution of ENRO in the muscle and hepatopancreas of *L. vannamei*, as well as the time needed after exposure to eliminate the antibiotic from the shrimp's tissue. This information will be helpful in defining the appropriate use of this antibiotic.

## 2. Materials and Methods 

### 2.1. Chemicals 

High purity (99.9%) ENRO and CIPRO (Riedel-de-HAEA, Vetranal, USA) were used in the analysis. The active ingredient enro-blend AQUA was prepared by Avimex Laboratory (Mexico) and was added to the shrimp feed by a food manufacturer to a final ENRO concentration of 200 mg/kg. Chromatographic-grade solvents were used for all analyses.

### 2.2. Animals 

This study was conducted with three replications, using healthy subadult white shrimp, *Litopenaeus* *vannamei* with weight to 15-16 g.

### 2.3. Physiochemical Parameters

The physiochemical parameters evaluated in the study, including temperature, dissolved oxygen (with an oximeter, YSI 55), pH (with a potentiometer, PHEP HANNA), and salinity (with a refractometer, Vitalsine SR6). Measurements were made at 4:00 and 16:00 h.

### 2.4. Experimental Design

The laboratory assay was conducted in the Laboratory of Marine Invertebrate Physiology, Research Center for Food and Development (CIAD), and a farm study was conducted on a commercial farm. The experiments were performed under similar conditions in Bahia de Kino Sonora, Mexico and each lasted 30 days.

#### 2.4.1. Laboratory Study

Six 450 L-capacity tanks were filled with natural seawater, and a closed recirculation system replaced the water daily. Each tank was stocked with 35 randomly selected shrimp, per 12 liters of water. Three of the tanks were treated with the ENRO feed. The other three tanks were used as controls, and shrimp in these tanks received a diet free of antibiotics. The tanks were connected to a water recirculation system, with a seawater concentration of 36‰, a 12-hour photoperiod, and a system to provide constant aeration.

Titanium heaters were used to maintain a constant temperature of 28 ± 1°C. Temperature, dissolved oxygen (oximeter YSI 55), salinity (Acuatic Eco-Systems), total ammonia (fotometer Ecosense, Mod.9500), and pH (potentiometer, Mettler Toledo, Seven Easy) were measured daily. The shrimp were fed three times a day, at 8:00, 13:00, and 18:00 h.

#### 2.4.2. Farm Study

This study was conducted on a shrimp farm in the state of Sonora, Mexico. Three ponds measuring 6 to 7 ha each was selected in different sections of the farm under the assumption that these ponds had not been previously given antibiotics. White shrimp (*L. vannamei*) was used to stock the ponds to a density of 32 shrimp per m^3^.

### 2.5. Feed

Before the studies were carried out, levels of ENRO were confirmed in the medicated and unmedicated feed. The extraction and chromatographic analyses for these tests was conducted using the methodology proposed by Houglum et al. [7].

The medicated feed was administered over a period of 14 days (treatment phase). Subsequently, the antibiotic-free diet was administered for 16 days (elimination phase). Shrimp samples were taken on days 1, 2, 4, 6, 8, 10, 12, and 14 during both the treatment and elimination stages, with an additional sampling at day 16 of the latter phase. Approximately 1 kg of shrimp was collected in the farm study, and 2 shrimps were collected from each tank in the laboratory study. The samples were stored at −20°C until they were analyzed.

### 2.6. Enrofloxacin Extraction from Shrimp Feed

A 0.5 g feed sample and 25 mL of acetonitrile mixture (0.02 M phosphoric acid at pH 3, 80 : 20, v/v) were vortexed (Baynstead Thermolyne Type 37600, USA) for 1 min. The tubes were then sonicated for 25 min (Cole-Parmer Mod 8895-DTH. Chicago, IL, USA) and mechanically stirred for 30 min at 34 × g (Kika Labortechnik KS501 DS1, Germany). Next, samples were centrifuged at 2054 × g and 15°C for 15 min (Beckman Coulter Allegra 6R. USA). The supernatant was filtered through glass fiber (Whatman GF/A), and the filtrate was collected in a 50 mL polypropylene tube. Fifty microliters of the filtered extract was adjusted to 10 mL with the mobile phase. Subsequently, the samples were injected into the liquid chromatograph, which was equipped with a fluorescence detector. The limit of quantitation (LQ) was 0.001 *μ*g/g for enrofloxacin and 0.01 *μ*g/g for ciprofloxacin.

### 2.7. Sample Preparation and Extraction Method of Enrofloxacin and Ciprofloxacin

#### 2.7.1. Shrimp Muscle Samples

Sampled shrimp were decapitated, and the cuticle was removed manually; the muscle was placed in properly labeled containers. Shrimp muscle was homogenized in a Phillips food processor (Mod.HR 2875/AM, Mexico). Subsequently, antibiotic analysis was done according to the methodology proposed by Kirbiš et al. [8], based on liquid-liquid extraction. This procedure consisted of weighing 2.5 g of shrimp muscle, to which 1.5 mL of Trizma Base solution (pH 9) was added. The tissue was homogenized with a glass rod followed by one min of vortexing (Baynstead Thermolyne Type 37600, USA), after which the mixture was allowed to stand for 15 min. 4 mL of reagent grade acetonitrile (J. T. Baker) was added, and the mixture was vortexed. The samples were sonicated for 15 min and centrifuged at 2054 × g for 30 min. The supernatant was removed with a Pasteur pipette and placed in a 15 mL centrifuge tube, after which the extraction was repeated with an additional 4 mL of acetonitrile to remove the supernatant and collect the extract. The supernatant was evaporated in a water bath at 45–50°C (VWR Scientific Products, Mod 1202, Series 180, USA) with constant airflow, to a total volume of 2.5 mL.

Fat was removed from the extract by adding 1.5 mL of reagent-grade hexane (J. T. Baker). The extract was then centrifuged at 2054 × g for 30 min at 15°C. The upper layer was discarded, and the process was repeated. The remaining aqueous phase was filtered using a syringe attached to an acrodisc (0.2 *μ*m; LC13 PVDF, Fisher Scientific, Pittsburgh, PA, USA). The extract was placed in a 4 mL amber vial for injection into the liquid chromatograph.

#### 2.7.2. Shrimp *Hepatopancreas* Samples 

The extracted hepatopancreas was homogenized, and ENRO and CIPRO analyses were performed per the technique described by Tang et al.  [1]. This method consisted of weighing 0.5 g of hepatopancreas in a 15 mL centrifuge tube and adding 1 mL of a mixture of 1 M NaCl : 0.2 M KH_2_PO_4_ (50 : 50, v/v) at pH 7.4; the mixture was then stirred and allowed to stand for 15 min. Next, 5 mL of reagent-grade dichloromethane (J. T. Baker) was added, and the mixture was stirred for 1 min and sonicated for 15 min. The samples were then centrifuged at 2054 × g for 30 min at 15°C. The lower layer of dichloromethane was removed by Pasteur pipette and transferred to a 15-mL glass centrifuge tube. An additional 5 mL of dichloromethane was added to each tube, and the samples were agitated in a mechanical shaker at 200 rpm for 5 min (Kika Labortechnik KS501 digital, Germany) and centrifuged for 20 min at 2054 × g and 15°C. The lower layer of dichloromethane was then removed and transferred to the tube containing the extract.

The extract was dried in a water bath at 45°C with continuous airflow. The extracts were reconstituted with 4 mL of a mixture of 1 M NaCl : 0.2 M KH_2_PO_4_ at pH 7.4 (50 : 50, v/v), stirred, and 4 mL of reagent-grade hexane was added and agitated for 1 min. This mixture was centrifuged at 2054 × g for 30 min at 15°C, and the top hexane layer was removed. This step was repeated once, and the extracts were combined. The extract was then filtered using a plastic syringe attached to an acrodisc (pore size 0.2 *μ*m). Subsequently, the extract was filtered and transferred to a plastic syringe attached to an acrodisc (pore size of 0.2 *μ*m). The extract was placed in an amber vial and stored until it was injected into the liquid chromatograph, which was equipped with a fluorescence detector.

The recovery rate for the extraction technique was 89.79 ± 11.88% for ENRO and 86.91 ± 9.86% for CIPRO.

### 2.8. Chromatographic Conditions

We used a VARIAN Mod 9010 liquid chromatograph coupled to a VARIAN Prostar 410 auto sampler (Varian Instruments Inc., Palo Alto, CA, USA) and a C18 column (Supelco Inc., Bellefonte, PA, USA, 5 *μ*m, 150 × 4.6 mm ID). The mobile phase consisted of 0.02 M phosphoric acid at pH 3 and acetonitrile (80 : 20, v/v), with an isocratic flow of 1 mL/min. A VARIAN Mod 9070 fluorescence detector (Varian Instruments Inc., Palo Alto, CA, USA), set to an excitation wavelength of 280 nm and emission of 440 nm, was also used.

### 2.9. Statistical Analysis

The data were analyzed using descriptive statistics. For calculation of the mean and standard deviation, we used the program NCSS [9].

## 3. Results 

### 3.1. Enrofloxacin Concentration in Shrimp Feed

**Figure 1:**
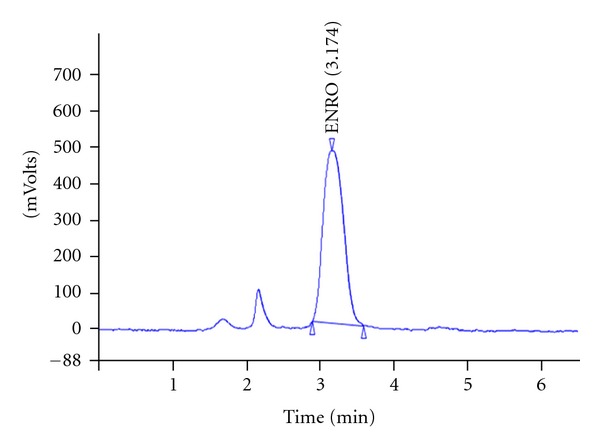
Liquid chromatogram of enrofloxacin (ENRO) in a sample shrimp feed (injection volume: 100 *μ*L, retention time 3.174 min).

Before the antibiotic-supplemented feed was given to the shrimp, ENRO levels in the feed were measured at 200.8 mg/kg by liquid chromatography (Figure 1). The retention time was 3.174 min, which matches the retention time of the ENRO standard used as a reference.

### 3.2. Physicochemical Parameters

In the laboratory bioassays, physical and chemical parameters were kept constant. The average tank salinity was 36.8 ± 0.8‰; average pH was 7.8 ± 0.1; temperature was maintained at 28.6 ± 0.2°C, and dissolved oxygen was 3.68 ± 0.8 mg O_2_/L.  Table 1 shows the average of the physicochemical parameters of each of the three ponds used in the farm study.

### 3.3. Concentration of Enrofloxacin and Ciprofloxacin in Shrimp Muscle Sampled in Laboratory and Farm Studies

#### 3.3.1. Enrofloxacin Concentration in Shrimp Muscle (Study in Laboratory and Farm)

**Figure 2:**
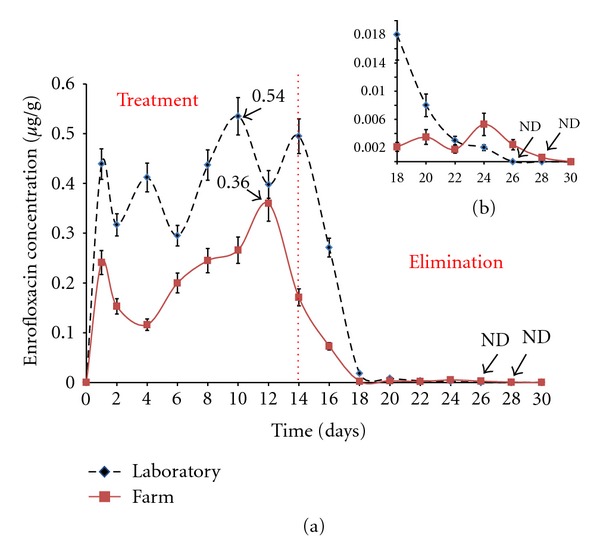
(a) Kinetic behavior of enrofloxacin (ENRO) in *L. vannamei* muscle.The dicontinuous line corresponds to the concentration of ENRO in the laboratory and the continuous line the concentration of antibiotics in the farm. (b) is an expansion of the graph corresponding to ENRO clearance time.

Figure 2(a) shows the kinetic behavior of ENRO in shrimp muscle under both laboratory and farm conditions, during the treatment and withdrawal stages. *C*_max⁡_ values for ENRO in muscle are shown in Table 2. The highest levels of antibiotic were detected 10 days after the initial application under laboratory conditions and 12 days after application in the farm study. Figure 2(b) shows the withdrawal time, which is the time required to achieve levels below the lowest measureable concentration (LQ) of the antibiotic (0.001 *μ*g/g for ENRO and 0.01 *μ*g/g for CIPRO). Non detected (ND) correspond to levels below at LQ. ENRO had a withdrawal time of 12 days under laboratory conditions and 14 days in the farm.

#### 3.3.2. Ciprofloxacin Concentrations in Shrimp Muscle (Study in Laboratory and Farm)

**Figure 3:**
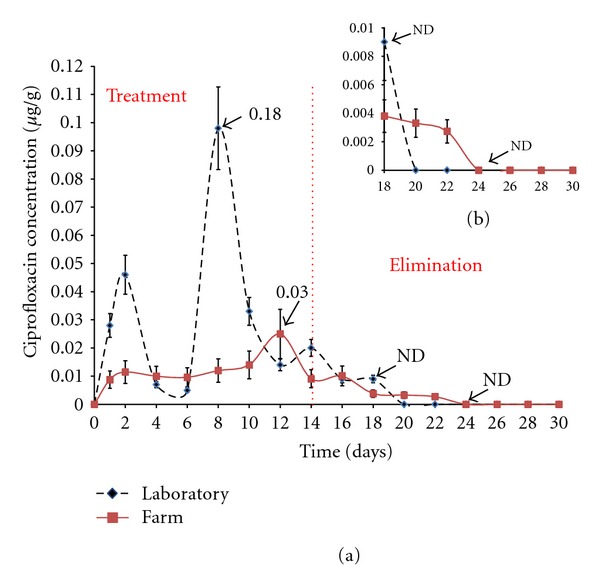
(a) Kinetic behavior of ciprofloxacin (CIPRO) in *L. vannamei* muscle. The dicontinuous line corresponds to concentration of CIPRO in the laboratory and the continuous line to concentration in the farm. (b) is an expansion of the graph corresponding to CIPRO withdrawal time.

Figure 3(a) shows the kinetic behavior of CIPRO in shrimp muscle in laboratory and farm during the treatment stage. Treatment-phase and elimination-phase CIPRO levels obtained under laboratory and farm conditions are shown in Table 2.

The greatest accumulation of CIPRO in the laboratory study was detected eight days after initial treatment, whereas the farm study required 12 days to reach maximum accumulation. In the elimination stage, the laboratory and farm withdrawal time based on the LQ (0.01 *μ*g/g) values were, respectively, 2 and 10 days (Figure 3(b)).

### 3.4. Concentration of Enrofloxacin and Ciprofloxacin in Hepatopancreas

#### 3.4.1. Enrofloxacin Concentration in Shrimp Hepatopancreas (Study in Laboratory and Farm)

**Figure 4:**
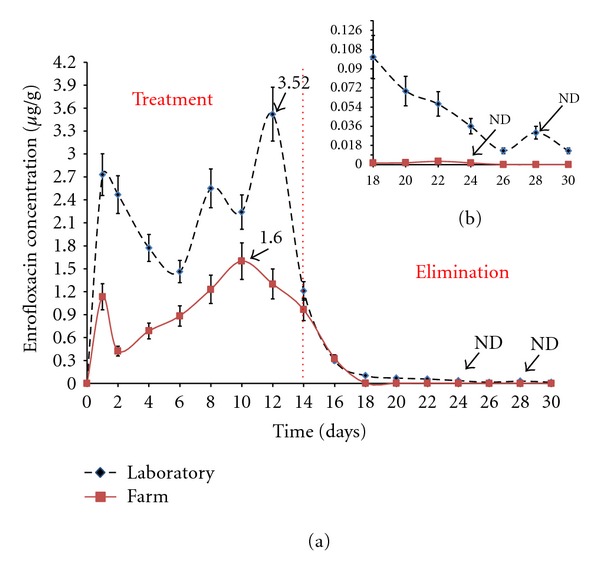
(a) Kinetic behavior of enrofloxacin (ENRO) in *L. vannamei* hepatopancreas. The dicontinuous line corresponds to concentration of ENRO in the laboratory and the continuous line to concentration in the farm. (b) is an expansion of the graph of ENRO withdrawal time.

Figure 4(a) shows the kinetic behavior of ENRO in the shrimp hepatopancreas under laboratory and farm conditions. ENRO and CIPRO accumulation values during ENRO therapeutic treatment through medicated feed for 14 days are shown in Table 2, with ENRO *C*_max⁡_ values reached at 12 days after the start of dosing. Under laboratory and farm conditions, the maximum ENRO level found was reached at day ten.

Figure 4(b) shows the elimination stage; under laboratory conditions, residual ENRO was still detected in the hepatopancreas at an average level of 0.013 ± 0.009 *μ*g/g 14 days after stopping treatment. In the farm, ENRO levels fell below the LQ 10 days after treatment.

#### 3.4.2. Ciprofloxacin Concentration in Shrimp Hepatopancreas (Study in Laboratory and Farm) 

**Figure 5:**
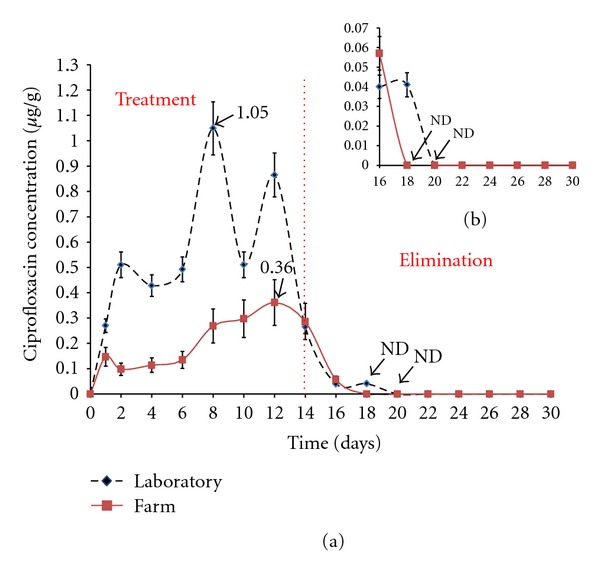
(a) Kinetic behavior of ciprofloxacin (CIPRO) in hepatopancreas of *L. vannamei* shrimp. The dicontinuous line corresponds to concentration of CIPRO in the laboratory and the continuous line to concentration in the farm. (b) is an expansion of the graph of CIPRO withdrawal time.

Figure 5(a) shows the kinetic behavior of CIPRO in the shrimp hepatopancreas in both laboratory and farm treatments. Under laboratory conditions, CIPRO Cmax was reached after eight days of treatment; under farm conditions, *C*_max⁡_ was reached after 12 days of treatment. Figure 5(b) shows the results for the elimination phase. The withdrawal time values for the laboratory and farm treatments were 6 and 4 days, respectively.

ENRO accumulation in muscle tissue was 33% lower in the farm study compared to the laboratory study and 55% lower in the hepatopancreas. The accumulated concentration of CIPRO was 66% lower under farm conditions than in the laboratory in both muscle and hepatopancreas tissue.

## 4. Discussion

### 4.1. Physicochemical Parameters

Salinity, pH, temperature, and dissolved oxygen (DO) influence the pharmacokinetic behavior observed in the laboratory and in the farm. Alzieu [10] showed that variations in salinity influence fundamental reproductive functions, such as, gametogenesis as well as nutrition and growth of aquatic species. Hunt [11] proposed that the ideal salinity for shellfish cultivation is 33‰. This value was approximately the average salinity maintained in the laboratory and farm studies (36.8 ± 0.8‰  and 36.4 ± 0.13‰, resp.). The variability in salinity values recorded may be due to latitude, climate, and, local hydrological characteristics.

Boyd [12] reported that most aquatic organisms tolerate a pH range of 6 to 9. The average pH in the farm and laboratory studies was 8.21 ± 0.06 and 7.8 ± 0.10, respectively, which is within the recommended range for shrimp cultivation. Extremely high or low pH conditions can kill over 50% of a shrimp population in the juvenile stage and dramatically reduce their movement through excess ammonia accumulation and the inability to transport oxygen [13]. Extreme pH values, combined with pollutants, such as, heavy metals, unionized ammonia, hydrogen cyanide, or hydrogen sulfide, can create a highly toxic environment. Swingle [14] reported that pH values below 4 and above 11 can cause fish death or at best, reduce survival rates and overall production [15].

For cultivated aquatic organisms, temperature is one of the most important parameters. Alzieu [10] reported that increased temperature increases metabolism, and that the extra energy requirements are met through the consumption of additional food. Extreme temperatures affect reproduction and survival of larvae. The ideal temperature is 28 ± 3°C [16]; the average temperature measured in the farm was within the recommended range, with morning temperatures averaging 29.26 ± 0.24°C and afternoon temperatures averaging 31.29 ± 0.30°C. In the laboratory, the temperature was kept constant at 28.6 ± 0.2°C.

Dissolved oxygen levels in the farm averaged 3.17 ± 0.30 mg O_2_/L in the morning and increased to 6.78 ± 0.89 mg O_2_/L in the afternoon. Alzieu [10] has reported that water has a lower oxygen concentration at night than in the morning, when photosynthesis provides a surplus of oxygen; this oxygen then dissipates into the atmosphere or is consumed during the process of respiration. Fry [17] noted that an aquatic organism can tolerate low oxygen levels for several hours without apparent effect of damage but may die if the condition is prolonged. Low oxygen levels make these organisms more susceptible to pests and diseases, and Plumb et al. [18] have reported that low oxygen content could affect an organism's ability to feed and grow.

Jiang et al. [19] studied the effect of different concentrations of dissolved oxygen on *L. vannamei* and found that the ideal level of dissolved oxygen is between 3.5–7.5 mg O_2_/L. If the level is less than 3.5 mg O_2_/L, the organism may develop hypoxia. In the laboratory, dissolved oxygen content remained constant at 3.68 ± 0.8 mg O_2_/L, which is within the recommended range. In addition, there was no decrease in the consumption of food by the organisms because of inadequate dissolved oxygen concentrations. In farm the levels were within the recommended range.

### 4.2. Enrofloxacin and Ciprofloxacin Accumulation

Measurement of antibiotic accumulation in shrimp tissues showed that the highest levels of ENRO and CIPRO were found under laboratory conditions. This difference is likely a result of the controlled, closely monitored physicochemical parameters, and diet in the laboratory. In the farm study, these conditions were more variable, and the cultured shrimp had alternative food sources, such as plankton. This discrepancy reflects the importance of considering extraneous variables by performing both study types.

Tu et al. [6] conducted a study using black shrimp (*Penaeusmonodon*) under farm and laboratory conditions; ENRO was administered throughout the study at a dose of 4000 mg/kg for 7 days. The average concentrations of ENRO in muscle tissue were 0.44 ± 0.27 *μ*g/g in the laboratory and 0.10 ± 0.01 *μ*g/g in the farm experiment, with rapid dissipation after treatment finished. This study, however, did not include a measurement of accumulated ENRO in the hepatopancreas and CIPRO concentration, even though CIPRO is reported to have antimicrobial activity. Furthermore, the discrepancy in accumulated ENRO between the farm and the laboratory was 22.7%, less than the 33% found in our study.

Several factors influence the kinetic behavior of antibiotics, including the cultivated species, the route of drug administration, the dosage used, and the rates of drug accumulation and elimination [3–6]. Xu et al. [3] reported that at an ENRO dose of 50 mg/kg administered to Chinese shrimp for 7 days, ENRO accumulation in muscle was 1.68 *μ*g/g and CIPRO accumulation was 0.07 *μ*g/g, and withdrawal time in muscle was 12 and 3 days for ENRO and CIPRO, respectively. Tu et al. [6] showed that a diet supplemented with 4000 mg/kg of ENRO in commercial L. vannamei ponds for 7 days led to lower levels of accumulated antibiotic in the muscle. This difference may be due to the lower bioavailability of the antibiotic in the marine environment. The seawater cations form complexes with quinolones reduce the capacity of shrimp to absorb the antibiotic. For this reason, a higher dose and longer treatment time are required when antibiotics are applied to marine organisms [20].

Another factor that influences the kinetic behavior of antibiotics is the individual response to the drug. When shrimp are sick or molting, they stop feeding, which reduces the accumulation of antibiotics in their systems. de Oliveira Cesar et al. [21] reported that shrimp more than one-month-old have molting cycles of 4.6 ± 0.5 days/cycle; at ages 3 and 6 months, the shrimp have molting cycles of 11.8 ± 1.7 days/cycle and 17.2 ± 2.7 days/cycle, respectively.

Other authors have proposed eliminating ENRO and CIPRO use in shrimp cultivation, based on clearance times between 3 and 12 days [3, 6]. These data are similar to those found in our study (2 to 14 days in the hepatopancreas and muscle). ENRO is cleared faster from the hepatopancreas than from the muscle, although ENRO and CIPRO reach higher concentrations in the hepatopancreas. This apparent discrepancy is due to the metabolic functions of the shrimp hepatopancreas, which are similar to those of the kidneys and stomach in other organisms [22], increasing metabolism and speeding drug clearance.

A comparison of antibiotic levels in shrimp tissue with the minimum inhibitory concentrations (MIC, 0.25 to 10 *μ*g/mL) for strains of Vibrio bacteria isolated from the same shrimp ponds [unpublished data] indicates that insufficient antibiotic was accumulated for bacterial inhibition. A study of the antimicrobial sensitivity of 144 strains of Vibrio isolated from shrimp culture systems showed that the bacteria had a MIC of 0.5 *μ*g/mL [23]. Mohney et al. [24] reported MIC values for the same antibiotic (0.45 *μ*g/mL) that were similar to those found in this study.

## 5. Conclusions

Although none of the fluoroquinolones are approved in the United States for use as aquaculture therapeutic agents, the potential for their extralabel use is of concern. The interest indicated by the aquaculture industry in these drugs and potential for the emergence of drug-resistant bacteria through their use have created a need for make studies about this compound. The generated date on concentration of ENRO and CIPRO in shrimp tissues can be used to help estimate if the use to this FQ is adequate in seawater, considering environmental factors of the region in the shrimp cultured.

## Figures and Tables

**Table 1:** Average physicochemical parameters in ponds at the research farm.

Physicochemical	Time of recording	
parameters	Morning	Afternoon
Temperature (^°^C)	29.26 ± 0.24	31.29 ± 0.30
Dissolved oxygen (mg O_2_/L)	3.17 ± 0.30	6.78 ± 0.89
Salinity (‰)	36.4 ± 0.13	—
pH	8.2 ± 0.06	—

*n* = 30.

—: not applied.

**Table 2:** Shrimp tissue enrofloxacin (ENRO) and ciprofloxacin (CIPRO) concentrations during each treatment stage in the laboratory and farm.

	Laboratory	Farm
	(*μ*g/g)	(*μ*g/g)
ENRO		
Muscle	0.54 ± 0.26	0.36 ± 0.17
Hepatopancreas	3.52 ± 1.90	1.60 ± 0.82
CIPRO		
Muscle	0.18 ± 0.13	0.03 ± 0.02
Hepatopancreas	1.05 ± 0.20	0.36 ± 0.08

*n* = 3.
